# Domestic Banks in Bangladesh Could Ensure Efficiency by Improving Human Resource Management Practices

**DOI:** 10.1371/journal.pone.0121017

**Published:** 2015-07-29

**Authors:** Abdul Kadar Muhammad Masum, Md. Abul Kalam Azad, Kazi Enamul Hoque, Loo-See Beh

**Affiliations:** 1 Department of Administrative Studies & Politics, Faculty of Economics and Administration, University of Malaya, Kuala Lumpur, Malaysia; 2 Department of Applied Statistics, Faculty of Economics and Administration, University of Malaya, Kuala Lumpur, Malaysia; 3 Department of Educational Management, Planning and Policy, Faculty of Education, University of Malaya, Kuala Lumpur, Malaysia; TNO, NETHERLANDS

## Abstract

The paper aims to examine the influence of human resource management (HRM) practices on bank efficiency using Malmquist index of total factor productivity. The model comprises HRM index that represents the quality of HRM practices. The results are decomposed into three efficiency scores, namely, technical efficiency, pure efficiency, and scale efficiency. In this study, panel data for 44 banks in Bangladesh are used for the period 2008-2013. This paper reveals that foreign banks are ahead in converting the influence of HRM practices into efficiency scores (0.946>0.833). On the other hand, domestic banks performed better than foreign banks in terms of pure efficiency and scale efficiency. But, in terms of technical efficiency, the domestic banks are regressed by 6.7% annually whereas foreign banks are progressed with a yearly value of 5.8%. The results are robust, because the Mann-Whitney test and Kruskall-Wallis test (non-parametric tests) also confirm the same results. This study emphasizes HRM practices in the banking industry to ensure efficiency in the long-term scenario. Domestic banks are suggested to ensure continuous development in HRM practices in order to compete with foreign banks.

## Introduction

In recent years, human resource management (HRM) practices are considered to be a key strategic partner in all organizations to attain critical business objectives [1]. Explicitly, achieving greater organizational performance (OP) and competitive advantage depend on the alignment of organizational strategies and HRM initiatives [2,3]. Banks play a vigorous role in the financial advancement of a nation. For appreciable bank performance, human resources are considered as prime business assets. But, quantifying their value is much more complex than that of other assets [4]. Knowledge and skill are indeterminate attributes of HR. It is hard to see their economic value in the organization because of their intangibility and latent nature. In this paper, the effect of HRM practices on the bank efficiency was examined in the context of Bangladesh. Here, suitable techniques and procedures from both HRM and economic theory were adopted to fill the present gap in the knowledge.

Bangladesh is a Next Eleven developing country in the world economy. Its total population is 159,147,170 placing it eighth globally [5]. The Bangladesh banking sector is one of the most prosperous industries in the Indian sub-continent, with foreign investors progressively paying more attention to grasp this sector. Therefore, there is an urgent need to look into the banking sector of Bangladesh. There are 62 banks with 8,794 branches operating in this country. Over the last three decades, Bangladesh has achieved noticeable success in accessing banking services. The population per branch was 57,700 and 20,162 respectively, in 2007 and 2010 [6]. Now, in 2014, the population per branch is 18,098. The statistics show that people are increasingly receiving banking services in the context of the number of branches. It is also evident that bank competition is increasing day by day. Unfortunately, most domestic banks of Bangladesh have been suffering from the insufficiency of managerial competence owing to professional HRM practices and the lack of expert manpower [7]. Hence, this study is a pioneer to examine the inevitability of investment in HRM for banks in Bangladesh to achieve the desired performance.

Organizational performance (OP) has been mostly measured in analyzing quantitative aspects (bank efficiency and performance) rather than quality aspect in the banking studies. To discover the connection between bank efficiency and management quality aspects, we implement a nonparametric frontier method of data envelopment analysis (DEA) developed by Färe,Grosskopf and Roos [8]. Here, we incorporate the HRM index to compute the Malmquist index for the banks in Bangladesh. In this model, we decompose the Malmquist index into 3 components: (a) pure efficiency, (b) shifts in production technology, and (c) scale efficiency. The present research investigates whether the efficiency growth in the banking sector of Bangladesh is endorsed to either technological change or technical efficiency change or both. Considering human resources as an integral factor of growth, this variable is incorporated in the Malmquist index calculation. Hence, the results of this research will enrich the quality of HRM practices, which needs to be integrated in the efficiency analysis for banking sector.

In our previous study [9], we examined the effect of HRM practices on bank performance in Bangladesh. A total 48 banks were evaluated over the period of 2004–2013. Findings of the previous paper were the mixed result of the impact of pre and post-world economic crisis in 2008 [10]. With additional criteria, this study mainly focuses on whether there is any change on the impact of post global economic crisis on bank performances. Additional criteria are: (i) increasing the number of input-output variables from 6 to 8 in order to achieve better discriminations in the efficiency results compared to our previous study which were not available for our previous study period (2004–2013) and; (ii) dropping 4 banks from our present study because of their consistent losses in the subsequent financial years. We assume that the incorporation of these problem banks may deviate the results since DEA is a relative measure of performance. Thus, our present study examines the impact of HRM practices on bank efficiency with better results compared to that of our previous effort [9]. In nutshell, this study wants to give insight the impact of post global economic crisis (2008) on bank performance especially developing countries like Bangladesh.

In this paper, section 2 briefly represents the overall scenario of the banking industry in Bangladesh. Section 3 compromises a succinct assessment of the existing literature regarding prime issues of HRM practices and efficiency measurement techniques to attain bank efficiency. The next section consists of variables and the data set. Section 5 contains the methodology that describes the suggested HRM component in Malmquist index decomposition. The outcomes of the investigation are described in Section 6. The findings of study, the shortcoming of the study and directions for future research are also deliberated in section 7 and 8 respectively. The last section contains the conclusion including suggestions for the banking sector.

## Banking Sector in Bangladesh

After the emergence of Bangladesh in 1971, Bangladesh Bank (BB)—the central bank of the country was established through Bangladesh Bank Order 1972. The banking industry started its journey with 2 state-run specialized banks (SDBs), 6 nationalized commercial banks (NCBs) and 3 foreign commercial banks (FCBs). The growth of the banking system in Bangladesh has moved through 3 stages. All the banks of Bangladesh were nationalized during the first phase of BB (1972–1982). Moreover, the credit focused on public investment and farming. Investment rates were decidedly controlled by the central bank and yearly inflation was 8.9% [7]. In 1972, the total deposits were 5.2 billion Takas where the NCBs held an 89.6% share of the total deposits. The deposits grew to 36.7 billion Takas including the 86.8% share of the NCBs in 1982. The BB had a legislating body including 9 members with a governor as its head for its 9 branches. In the second phase of reforms, which lasted from 1983 to1989, the NCBs started to be privatized (private commercial banks–PCBs) by the government regulation of banks. The deposits’ share of NCBs reduced to 64% of the total deposits (164.6 billion Takas) in 1989, and that of the PCBs increased from 0.33% to 24.4% during the years (1982–1989). At that time, inflation averaged 9.3% [11]. The third and current phase started in 1990 when PCBs were merged and additional monetary liberalization happened. By 2014 (January-March), the total deposits were 6105.4 billion Takas. The deposits’ share of PCBs developed to 63.18%, while that of the NCBs tumbled to 25.57%. During this phase, inflation averaged 6.96%.

In the present day, the banking sector of Bangladesh can be categorized into 5 groups. Group 1: There are 4 State Owned Commercial Banks (SOCBs). These banks, together, control more than 54% of total deposits. These are majorly or fully owned by the Bangladesh government. Group 2: There are 5 Specialized Banks (SDBs) that are established for special purposes, such as industrial or agricultural development. SDBs are also majorly or fully owned by the Bangladesh government. Grameen Bank, a Nobel-prize winner, is a specialized micro-finance institution. It is the pioneer of micro-credit concept worldwide and have made a considerable contribution towards the empowerment of women and reduction of poverty in Bangladesh. Group 3: Private Commercial Banks (PCBs) are the highest growth sector due to better service and products. There are 39 PCBs in Bangladesh. The 31 conventional PCBs perform banking in interest-based operations. There are also 8 PCBs that are performing banking based on Islamic Shariah (e.g., Profit-Loss sharing mode). Group 4: There are 9 Foreign Commercial Banks (FCBs). The branches of these foreign banks are unified with the overseas branches. Group 5: There are also 4 non-scheduled banks. These are Probashi Kollyan Bank, Karmashangosthan Bank, Jubilee Bank, and Ansar VDP Unnayan Bank.

As of 2014 (June), the total number of bank branches was 8,794. Among these branches, 57.11% (5,022) are situated in the rural areas and 42.89% (3,772) are situated in the urban regions. The SOCBs hold 3,536 branches, SDBs hold 1,496 branches, PCBs hold 3,692 branches, and FCBs hold 70 branches (source: BB website at http://www.bb.org.bd/). All commercial banks in Bangladesh provide savings account, current account, tele-banking, internet banking, home-banking, and SWIFT account service as like commercial banks in other countries.

## Literature Review

Nowadays, human resources are the most valuable and dynamic assets of all organizations [4]. They require considerable care from the management to show their maximum capacity in their work. Most researchers identified a significant relationship between HRM practices and OP [12]. A good number of research was conducted to demonstrate the relationship between OP and HRM practices. To describe this relationship, researchers categorized the existing studies in two clusters: first, studies concerning the influence of some selected HRM practices on OP, such as recruitment [4,13], employee participation [14], training and development [15–17], compensation and rewards [18–20], and employee relations [21]; second, studies on the effect of HRM practices on OP [12,22–26]. These studies assessed the effect of HRM practices on OP applying various traditional approaches within different industries. Most of the researchers identified a significant relationship between HRM practices and OP. Moreover, banks can earn distinctive financial gain by practicing HRM efficiently and effectively [25].

A study on a large organization conducted by Wright, Gardner, Moynihan, Allen [23] observed that HR practices were intensely connected to future performance of organizations in USA and Canada. An empirical study of 200 Vietnamese small medium enterprises (SMEs) found that incentive compensation, preparation, and performance appraisal have positive effects, with incentive compensation having the highest impact on employee performance [27]. Similarly, in a survey of 236 operational managers of steel firms in Taiwan, Lee, Lee and Wu [24] revealed that HRM practices–HR planning, training and development, incentives, performance appraisal, teamwork, and employment security–were positively related to OP. One more research on HRM practices in large firms in the US indicated that OP was positively correlated with HRM practices [28]. This paper focused on 4 HRM practices suggested by Dessler and Varkkey [29] presuming that these 4 HRM practices have a noteworthy influence on OP.

According to Dessler and Varkkey [29], recruitment and selection are the first and crucial determinants in HRM practices to acquire the requisite employees for the organization [4,13]. Some researchers revealed that efficiency has a connection with both employee selection and employee training [30,31]. Recruitment is a practice for attracting competent applicants to get the right person(s) at the right place. Boohene and Asuinura [32] observed that employee corporate performance was positively related to effective recruitment and selection practices, and negatively to performance appraisal practices in Ghana. In a study of 178 manufacturing companies in Greece, Katou and Budhwar [33] found that OP was positively related to recruitment practice, training practice, and employee relations practice. Lopez, Peón and Ordá [34] examined organizational learning with 4 HRM practices–recruiting, decision-making, training, and compensation. They concluded that organizational learning was positively influenced by employee participation in decision-making, selective hiring, and effective training. Since employee turnover is an unacceptable and costly issue, management should pay attention to strategic HR planning, as it is an effective selection process to attract talented employees.

Secondly, training and development is one of the key functions of HRM practices to meet changing demands in organizations. It is an important variable in organizational success [22, 26]. It is a systematic development of one’s knowledge and skill to execute a given job. Lopez,Peón and Ordás [34] identified training as a key element to attain competencies that, ultimately, improve OP. Some researchers stated that adequate training programs for employees develop a sense of emotional sensitivity and commitment to their tasks [16,35]. In contrast, Haines III, Jalette and Larose [36] claimed that employee turnover increases because of training and development. Zheng and Lamond [37] suggested training as a significant factor of employee retention. A study on Moroccan firms was conducted by Dumas and Hanchane [15] to evaluate the impact of training programs on OP. They reported that training programs boost the performance of Moroccan firms and it was identified as an essential part of a HR development strategy. Besides, Katou [17] found strongly positive relation between OP and the development of human capital in the Greek manufacturing sector.

Thirdly, compensation is also a vital function in HRM practices to satisfy the employees. Researchers found a positive influence of HR compensation function on OP [19]. Additionally, different types of compensation packages develop sense of loyalty among current employees and increase employee productivity. Consequently, the employee turnover rate is reduced [38]. Dessler and Varkkey [29] defined compensation as any type (direct or indirect) of financial reward or payment going to staffs arising from their services. A survey on HRM practice was conducted by Sabeen and Mehbob [18] in Pakistan. Five hundred participants from 18 private banks participated in this survey. They identified financial incentives as a significant determinant for organizational success. Bakshi, Mathur, Bhagat, Kalyankar [39] noted that impartial, transparent, and unbiased competitive compensation as well as reward system encouraged skilled employees to stay for a long period in an organization. In support of this, de Moraes, Godin, Tamaoki, Faloppa, Bhandari, Belloti [19] reported that strategic compensation policies enhanced individual performance. Subsequently, it strengthens the business strategy. Dreher and Dougherty [40] also encouraged reward-based motivation to enhance individual performance in organizations. A study on China conducted by Peng, Jiang, Zhang, Xiao, Song, Feng, et al. [41] found that employees realize a sense of commitment to an organization in respect of its strategic compensation practices and that the intention to leave decreases in such organizations.

Fourthly, employee relations have been recognized to be a communication process. It provides fair, transparent and justified treatment of employees to equip them with the knowledge, behavior, and attitude [42]. In addition, good employee relations decrease the risk of employee turnover. This process also helps managers to achieve enhanced leadership and supervisory quality. Cho and Erdem [43] reported that professionals and academicians only pay attention to the recruitment and selection practices, training practice, and compensation practice. But, the effect of employee relations on a firm’s performance has not been comprehensively investigated. They also stated that employee relationship problems were the highest concern of most HR managers. Umene-Nakano, Kato, Kikuchi, Tateno, Fujisawa, Hoshuyama, et al. [44] suggested the need for a strategic approach to employee relations to achieve organizational success. They stated that a positive employee attitude and commitment increase competitive advantage and enhance OP. A study on employee relations conducted by Gittell,Von Nordenflycht and Kochan [45] revealed that workplace justice influenced employees’ attitude including, loyalty, job satisfaction, organizational commitment, and job performance. McAuliffe, Daly, Kamwendo, Masanja, Sidat, de Pinho [46] stated that good employee relations can ascertain through improving employees’ participation and involvement in administrative decision-making, union relations, cash profit-sharing program, and employees’ safety and security. Therefore, organizations can implement a proactive strategy of investing in employee relations to increase employees’ loyalty and attract qualified human capital. Consequently, customer satisfaction will improve through service quality, which affects OP.

At present, the service quality of banks is a foremost factor to survive in banking markets due to the domestic and global competition. Previous studies show that employee competences have a positive correlation with service quality [4,47]. Consequently, to achieve the quality services in banking, there are some important preconditions: placement of the right people with skills, abilities and right knowledge of banking services, motivated employees to spend their skills, abilities and knowledge in offering services to clients. To fulfill the above mentioned conditions, large investment in quality of HRM practices is essential. For justification of this investment, the relation between bank efficiency and HRM practices should be explained. The prime thrust of this paper is to endorse the HRM practices as a quality component by validating and analyzing critical relationships between bank efficiency and HRM practices.

Previous literature shows that inadequate research exists to examine the rapport between the HRM practices and OP in the banking sector of Bangladesh. The literature on bank performance has been rapidly enriched over the last decades. A number of studies have used parametric models for analyzing efficiency. But, some authors have identified having inherent difficulties in parametric models regarding the assumptions for model fitting [48–50]. For avoiding such problems, data envelopment analysis (DEA)—a nonparametric model may be applied for superior efficiency estimate without any presuppositions. Here, input/output variables are in invariant form. Notably, DEA works on panel data and it can assess the efficiency of business units in a technical form. In this paper, we used a DEA model suggested by Färe and Grosskopf [51] incorporating the HRM quality index as a quality component for achieving efficiency among the banks. The quality index suggested by De Nicola, Gitto and Mancuso [52] was calculated in the DEA model. The literature review of the study explains the rationality of integrating HRM practices in bank efficiency measurement. The findings of the study will provide top level manager a clear insight to pay special attention for HRM practices in banking sector.

## Data and Variables

The variables of this study are designed from the background work by Chang and Huang [13]; Chou and Chang [53]; Somarriba and Pena [54]; Li,Liu and Meng [55] and De Nicola, Gitto and Mancuso [52]. We use 5 variables for input, i.e., X_1_: total branches, X_2_: total employees, X_3_: total operating expenses, X4: total loanable assets, and X5: total deposits. We also use 3 variables for output, i.e., Y1: total loans, Y2: net operating income, and Y3: total number of transactions. Here, our sample size is 44 as decision making units (DMUs). Data are collected from BankScope data base from 2008 to 2013. In this study, an overall quality index of HRM is achieved through nominated 4 indicators; i.e., annual HR recruitment (HRRec), HR compensation budget (HRCom), HR training and development budget (HRTrD), and employee relations budget (HREre). Table 1 shows summary statistics including the variables used in this research.

**Table 1:** Summary statistics of Data, 2008–2013.

Variables	Min	Max	Mean	Variation Coef.
Input (X_i_)				
X_1_: Total branches	48	816	96	13.812
X_2_: Total employees	3016	28096	7145	14.623
X_3_: Total operating expenses	137	319	158	8.197
X_4_: Total loanable assets	1485	154231	4865	16.752
X_5_: Total deposits	1036	175123	5235	45.234
Output (Y_i_)				
Y_1_: Total loans	985	12041	2563	19.453
Y_2_: Net operating income	38.5	782.5	98.7	7.078
Y_3_: Total number of transactions	981227	2054861	1325486	213.759
HRM index				
*HRRec*(annual)	45	210	76	0.095
*HRTrD*(million)	14.8	42.5	21.2	0.823
*HRCom*(million)	75	210	102.5	1.453
*HREre*(million)	20	53.10	20.09	0.612

Source: Bank annual report (2008–2013)

## Methodology

In the recent years, extensive use of DEA has been employed for performance evaluation. The reason behind is that a DEA model can convert multiple inputs/outputs in performance index ignoring the uncovered relationship of data among the DMUs [56]. Moreover, pre-assumptions are not required to operate the model, which motivates researchers to use DEA more than other methods [57]. We also use the Malmquist index that was developed by Malmquist [58] and Caves,Christensen and Diewert [59].

### Malmquist index of total factor productivity and quality components

In this study, the methodology is motivated from our earlier study [9]. If the input vector is represented by (*x* ^*t*^), and the output vector by (*y* ^*t*^), the production technology can be defined as:

$$P^{t}=\left[\left(Y^{t},A^{t},X^{t}\right):\;X^{t}\;can\;produce\;Y^{t}\;with\;a\;quality\;level\;of\;A^{t}\right]$$ (1)

According to Chou, Shao and Lin [60] and Coelli, Rao, O'Donnell, Battese [61], the notion of disposability of inputs and outputs is satisfied by this production set. A distance function of Eq 1 can be demonstrated with the guidelines of Shepard [62] as follows:

$${\text{D}}_{i}^{t}\left(y^{t},a^{t},x^{t}\right)=\text{Sup}\left(\lambda :(x^{t}/\lambda ,y^{t},a^{t})\in S^{t}\right)$$ (2)

Here, *λ* stands for the radial factor that adjusts the output vector’s position. We can duplicate Eq 2 as linear programming (LP) converting for a DMU;

$$\begin{array}{rr} {\left(D\left(y^{t’},a^{t\prime },x^{t’}\right)\right)}^{-1}=\text{min}\;\lambda :\;\sum_{t=1}^{T}z^{t}x^{tn}≦\lambda x^{t’n}, & n=1,\ldots \ldots \ldots ,N\\ \sum_{t=1}^{T}z^{t}y^{tm}≧xy^{t’m}, & m=1,\ldots \ldots \ldots ,M\\ \sum_{t=1}^{T}z^{t}a^{tj}≧a^{t’j}, & j=1,\ldots \ldots \ldots ,J\\ z^{t}≧0, & t=1,\ldots \ldots \ldots ,T \end{array}$$ (2.1)

Here, the intensity variable is *z* ^*t*^.

Considering two consecutive time frames (e.g., t and t+1) and combining the distance function of Eq 2, the Malmquist index can be shown as follows:

$$MI=\;M_{i}^{t,t+1}\left(y^{t+1},a^{t+1},x^{t+1},y^{t},a^{t},x^{t}\right)=\sqrt{\frac{D_{i}^{t}\left(x^{t+1},a^{t+1},x^{t+1}\right)}{D_{i}^{t}\left(y^{t},a^{t},x^{t}\right)}\times \frac{D_{i}^{t+1}\left(y^{t+1},a^{t+1},x^{t+1}\right)}{D_{i}^{t+1}\left(y^{t},a^{t},x^{t}\right)}}$$ (3)

At this point, we include the quality index $\left[\sqrt{\frac{A_{i}^{t}\left(a^{t+1}\right)A_{i}^{t+1}\left(a^{t+1}\right)}{A_{i}^{t}\left(a^{t}\right)A_{i}^{t+1}\left(a^{t}\right)}}\right]$ proposed by Färe, Grosskopf, and Roos [8] within the Eq 3 and decomposed into 3 main elements–technical efficiency changes *(Eff)*, technical changes *(Tech)*, and quality index (*QI*).

The Malmquist index of total factor productivity is expected to be liable for the quality issue in the output function as all 3 elements are in a multiplication form. Eq 4 indicates the decomposition.

$$MI=\sqrt{\frac{A_{i}^{t}\left(a^{t+1}\right)A_{i}^{t+1}\left(a^{t+1}\right)}{A_{i}^{t}\left(a^{t}\right)A_{i}^{t+1}\left(a^{t}\right)}}\times \frac{D_{i}^{t+1}\left(y^{t+1},x^{t+1}\right)}{D_{i}^{t}\left(y^{t},x^{t}\right)}\times \sqrt{\frac{D_{i}^{t}\left(y^{t+1},x^{t+1}\right)}{D_{i}^{t+1}\left(y^{t+1},x^{t+1}\right)}\times \frac{D_{i}^{t}\left(y^{t},x^{t}\right)}{D_{i}^{t+1}\left(y^{t},x^{t}\right)}}$$ (4)

Here,

$$Quality\;index\left(QI\right)=\sqrt{\frac{A_{i}^{t}\left(a^{t+1}\right)A_{i}^{t+1}\left(a^{t+1}\right)}{A_{i}^{t}\left(a^{t}\right)A_{i}^{t+1}\left(a^{t}\right)}}$$ (5)

$$Technical\;efficiency\;change\left(Eff\right)=\left[\frac{D_{i}^{t+1}\left(y^{t+1},x^{t+1}\right)}{D_{i}^{t}\left(y^{t},x^{t}\right)}\right]$$ (6)

$$Technical\;change\left(Tch\right)=\sqrt{\frac{D_{i}^{t}\left(y^{t+1},x^{t+1}\right)}{D_{i}^{t+1}\left(y^{t+1},x^{t+1}\right)}\times \frac{D_{i}^{t}\left(y^{t},x^{t}\right)}{D_{i}^{t+1}\left(y^{t},x^{t}\right)}}$$ (7)

So,

$$Malmquist\;Index\;with\;Quality\;\left(MI\right)=Eff_{\left(t,t+1\right)}\times Tch_{\left(t,t+1\right)}\times QI_{\left(t,t+1\right)}$$ (8)

Note that, a value of 1 explains that the efficiency remains equal compared to the period (t) in (t+1). Again, a value of more than 1 represents progress and less than 1 indicates a regress in efficiency as a relative measure.

Eq 4 is a constant return to scale (CRS), which means a change in input will cause a proportional change in output. With connection to the study requirements, this needs to switch to the variable returns to scale (VRS) method, as shown in Eq 9.

After further decomposition of *Eff*, Eq 6 is shown below:

$$Eff=\left[\frac{D_{i}^{t+1}\left(y^{t+1},x^{t+1}\right)}{D_{i}^{t}\left(y^{t},x^{t}\right)}\right]=\left[\frac{D_{VRS}^{t+1}\left(y^{t+1},x^{t+1}\right)}{D_{VRS}^{t}\left(y^{t},x^{t}\right)}\right]\;\left[\frac{D_{i}^{t+1}\left(y^{t+1},x^{t+1}\right)}{D_{i}^{t}\left(y^{t},x^{t}\right)}\times \frac{D_{VRS}^{t}\left(y^{t},x^{t}\right)}{D_{VRS}^{t+1}\left(y^{t+1},x^{t+1}\right)}\right]$$ (9)

Here, *D*_*VRS*_ is the output distance function for variable returns to scale. The initial part of the Eq 9 is termed as pure efficiency (PE). PE describes the pure change in technical efficiency from the time t to t+1. PE is the change in efficiency due to technology. The remaining change of efficiency is named as scale efficiency (SE)–changes in effect due to economies of scale. Thus,

$$Pure\;efficiency\;Changes\;\left(PE\right)=\left[\frac{D_{VRS}^{t+1}\left(y^{t+1},x^{t+1}\right)}{D_{VRS}^{t}\left(y^{t},x^{t}\right)}\right]$$ (10)

$$Scale\;efficiency\;changes\;\left(SE\right)=\left[\frac{D_{i}^{t+1}\left(y^{t+1},x^{t+1}\right)}{D_{i}^{t}\left(y^{t},x^{t}\right)}\times \frac{D_{VRS}^{t}\left(y^{t},x^{t}\right)}{D_{VRS}^{t+1}\left(y^{t+1},x^{t+1}\right)}\right]$$ (11)

Combining Eqs 5 and 9, it comes as the product of *QI*, *Tech*, *PE* and *SE*. Now, Eq 8 can be expressed as follows:

$$Malmquist\;index\;with\;Quality\;\left(MI\right)=QI_{\left(t,t+1\right)}\times Tch_{\left(t,t+1\right)}\times PE_{\left(t,t+1\right)}\times SE_{\left(t,t+1\right)}$$ (12)

We duplicate Eq 2.1 as linear programming (LP) for calculating HRM practices index *a* ^*t*^;

$$\begin{array}{rr} {\left(D_{i}^{0}\left(x^{t’,0},\;y^{t’,0},a^{t^{\prime },0}\right)\right)}^{-1}=\text{min}\;\lambda :\;\sum_{t=1}^{T}z^{t,0}x_{n}{}^{t,0}≦\lambda x_{n}{}^{t’,0}, & n=1,\ldots \ldots \ldots ,N\\ \sum_{t=1}^{T}z^{t,0}y_{m}{}^{tm,0}≧y_{m}{}^{t’m,0}, & m=1,\ldots \ldots \ldots ,M\\ \sum_{t=1}^{T}z^{t,0}a_{j}{}^{tj,0}≧a_{j}{}^{t’j}, & j=1,\ldots \ldots \ldots ,J\\ z^{t,i}≧0, & t=1,\ldots \ldots \ldots ,T \end{array}$$ (13)

The technique for calculating HRM practices (*a*) alone, the following LP can be used for the Eq 5:

$$\begin{array}{rr} {\left({\hat{D}}_{i}^{0}\left(x^{t’,0},\;y^{t’,0}\right)\right)}^{-1}=\text{min}\;\lambda :\;\sum_{t=1}^{T}z^{t,0}x_{n}{}^{t,0}≦\lambda x_{n}{}^{t’,0}, & n=1,\ldots \ldots \ldots ,N\\ \sum_{t=1}^{T}z^{t,0}y_{m}{}^{tm,0}≧y_{m}{}^{t’m,0}, & m=1,\ldots \ldots \ldots ,M\\ z^{t,i}≧0, & t=1,\ldots \ldots \ldots ,T \end{array}$$ (14)

Using Eq 14, both QI (*a* ^0^) and QI (*a* ^1^) need to be calculated.

### HRM quality index

In the above model, the quality index *QI*_(*t*,*t+1*)_ requires a single value that is used in Eqs 5 and 12. We use a regression model to compress the nominated 4 HRM practices indicators into one variable, namely, OvHR—overall HRM indicator. Similar studies of Li, Liu and Meng [55]; Masum, Azad and Beh [9]; Chou and Chang [53]; and De Nicola, Gitto and Mancuso [52] have also encouraged us to develop the following regression model to calculate OvHR for this study.

$${\text{OvHR}}_{t}=w_{0}+w_{1}\left(\frac{1}{HRRec}\right)+w_{2}\left(\frac{1}{HRTrD}\right)+w_{3}\left(\frac{1}{HRCom}\right)+w_{4}\left(\frac{1}{HREre}\right)+\varepsilon_{t}$$ (15)

Here,*w*_0_ is the intercept;*w*_1_,*w*_2_,*w*_3_,*w*_4_ are the parameters of the equation, and statistical error is denoted by *ε*_*t*_. Here, OvHR indicates the quality aspects of HRM practices when the value of regression is at satisfactory level. In contrast, OvHR represents less or no significance in the calculation of the OPs for poor regression value. For the said calculation, statistical package R (FEAR library) was employed.

## Results

### HRM quality index

In the first section of the empirical results, using the Eq 15, we determine the association between essential 4 HRM practices indicators with OvHR. Here, the pooled-OLS (ordinary least squares) method is used [63] on our panel data. In Table 2, the obtained results are summarized.

**Table 2:** Estimated regression model for OvHR.

	Estimate	Std. error	t statistics	p-Value
*w*_0_	0.975	0.078	11.852	0.000
1/*HRRec*	0.523	0.041	0.516	0.000
1/*HRTrD*	0.727	0.065	0.428	0.014
1/*HRCom*	0.645	0.034	1.335	0.001
1/*HREre*	0.408	0.043	0.452	0.012

Note: R^2^- 0.284, F-statistic- 0.745 and P-value 0.009

Table 2 reveals that HRRec and HRCom are the two significant variables. Factor analysis is run to combine the 4 HRM practices indicators in OvHR. First, in Table 3, the association values are computed and shown in Pearson correlation matrix. Now, factorial analysis, using the varimax rotation model, is exercised with obtained results from Table 3. The results of factorial models are summarized in Table 4.

**Table 3:** Pearson correlation matrix.

	*1/HRRec*	*1/HRTrD*	*1/HRCom*	*1/HRToR*
1/*HRRec*	1			
1/*HRTrD*	0.301	1		
1/*HRCom*	0.421	0.258	1	
1/*HREre*	0.211	0.287	0.175	1

**Table 4:** Factorial analysis of OvHR.

	Factor loading	Uniqueness
1/*HRRec*	0.843	0.950
1/*HRTrD*	0.484	0.904
1/*HRCom*	0.855	0.950
1/*HREre*	0.451	0.807

### Malmquist index of total factor productivity

In the second section of the empirical results, Table 5 demonstrates the summary results obtained from Eqs 4 and 12. In this study, all the values are in the form of mean statistics over the period of 2008–2013. Table 5 summarizes the upper and lower scores for all the dimensions of the efficiency changes of sample banks. Compared to our previous study [9], the efficiency scores are significantly discriminate among the examined banks.

**Table 5:** Summary results of Malmquist index (MI) and its decomposition.

Domestic Banks	MI	QI	Tch	PE	SE	Foreign Banks	MI	QI	Tch	PE	SE
AB Bank	0.913	0.958	0.856	0.965	1.154	Bank Alfalah	0.596	0.897	0.897	0.986	0.752
BCBL	0.846	0.895	0.895	0.958	1.102	Citibank NA	1.213	1.197	0.917	1.186	0.932
Bank Asia	1.073	0.919	1.219	0.963	0.995	CBCL	0.719	0.795	0.958	0.986	0.957
BRAC Bank	1.019	0.956	0.963	0.956	1.158	Habib Bank	0.698	0.819	0.963	0.916	0.966
Dhaka Bank	0.696	0.897	0.956	1.236	0.657	HSBC Ltd	1.075	0.975	0.956	0.916	1.259
DBBL	1.166	0.895	0.936	1.195	1.165	NBP	0.991	0.975	1.236	0.867	0.949
EBL	0.537	0.819	0.895	0.819	0.895	SCB	1.197	1.207	1.356	0.757	0.966
FBL	0.345	0.575	0.819	0.895	0.819	SBI	0.863	0.823	1.312	0.945	0.846
IFIC Bank	0.515	0.675	0.956	0.819	0.975	Woori Bank	0.672	0.824	0.924	0.985	0.895
Jamuna Bank	0.820	0.745	0.897	1.259	0.975						
Meghna Bank	0.943	0.986	0.895	0.949	1.127						
MBL	0.891	0.886	0.819	1.259	0.975						
Midland Bank	0.646	0.716	0.975	0.949	0.975						
Modhumoti Bank	0.762	0.895	0.975	0.956	0.914						
MTBL	0.574	0.819	0.845	0.897	0.924						
NBL	0.641	0.687	0.986	0.895	1.058						
NCC Bank	0.790	0.865	0.986	0.819	1.131						
NRB Bank	0.595	0.895	0.916	0.975	0.745						
NRB CB	0.722	0.819	1.059	0.975	0.854						
NRB Global	0.603	0.675	0.949	1.245	0.756						
One Bank	0.714	0.775	0.975	0.986	0.958						
Prime Bank	0.788	0.827	0.927	0.986	1.043						
SBL	0.752	0.895	0.975	0.963	0.895						
Standard Bank	0.625	0.819	0.975	0.956	0.819						
CBL	0.887	0.975	0.927	1.006	0.975						
TBL	0.892	0.897	0.975	0.819	1.245						
UCBL	0.839	0.895	0.975	0.975	0.986						
UBL	0.730	0.819	0.927	0.975	0.986						
ALFL	0.650	0.795	0.963	0.927	0.916						
EXIM Bank	0.662	0.775	0.956	0.975	0.916						
FIBL	0.629	0.805	0.936	0.975	0.856						
IBBL	1.017	0.963	0.956	1.156	0.956						
SJIBL	0.617	0.956	0.825	0.836	0.936						
SIBL	0.657	0.936	0.817	0.981	0.876						
UBL	0.593	0.875	0.756	0.945	0.949						
Geometric Mean	0.747	0.845	0.933	0.984	0.962		0.891	0.946	1.058	0.949	0.947

Table 6 summarizes the upper and lower scores for all the dimensions of the efficiency changes.

**Table 6:** Summary statistics of Malmquist index (MI).

Domestic Banks	MI	QI	Tch	PE	SE	Foreign Banks	MI	QI	Tch	PE	SE
Min	0.345	0.486	0.756	0.819	0.657		0.596	0.795	0.897	0.757	0.752
Max	1.166	0.986	1.219	1.259	1.245		1.213	1.207	1.356	1.186	1.259
Mean	0.732	0.833	0.933	0.979	0.960		0.891	0.946	1.058	0.949	0.947
% change	-26.8%	-16.7%	-6.7%	-2.1%	-4%		-10.9%	-5.4%	5.8%	-5.1%	-5.3%

### Robustness of results

In the last section of the empirical results, we test robustness of the derived data from DEA calculation in two different independent sample groups (domestic and foreign banks). Coakes and Steed [64] demonstrated that with an even sample distribution, the Mann-Whitney test is the relevant test. This method of testing robustness is designed from the past research of Isik and Hassan [65] along with Sufian, Muhamad, Bany-Ariffin, Yahya, Kamarudin [66].

The parametric t-test results show that foreign banks are scored 0.946 whereas domestic banks are marked with 0.833 in Table 7. In terms of the total efficiency test, domestic banks scored 0.732 as efficient and the foreign banks are scored efficient with a value of 0.891. The Mann-Whitney test and Kruskall-Wallis test as shown in Table 7 are significant at either the 1% or 5% level.

**Table 7:** Robustness of results.

	Test groups					
	Parametric test		Non-parametric tests			
	t-test		Mann-Whitney test		Kruskall-Wallis test	
Test statistics	t(Prb>t)		z(Prb>z)		X^2^(prb> X^2)^	
	Mean	t	Mean rank	z	Mean rank	X^2^
Quality indicator (QI)						
Domestic Banks	0.833	-1.245**	56.09	-2.142***	56.09	4.581***
Foreign Banks	0.946		61.31		61.31	
Total efficiency (MI)						
Domestic Banks	0.732	-2.475***	38.54	-1.925**	38.54	3.705***
Foreign Banks	0.891		47.36		47.36	

Note:

** and *** indicate significance level at the 5% and 1% levels respectively.

## Discussions

The major findings of this research are categorized in three clusters. First, Tables 2, 3 and 4 refer the construction of the first part of our proposed model. We compress the 4 HRM practices (HRRec, HRTrD, HRCom and HREre) in a single variable called OvHR. The results from Table 2 reveal that only HRCom and HRRec are significant (among the 4 nominated indicators) at the 5% level. Hence, none of the nominated indicators of HRM practices are exclusively associated to OvHR. From Table 3, the correlation matrix indicates a moderate level of relationship within the nominated quality indicators of HRM. Next, the summary results from Table 4 reveal that the variance of HRRec and HRcom varies at 0.05% level, followed by HRTrD and HREre with 0.096% and 0.193% variance respectively.

Second, Table 5 shows that only 8 banks (out of the 44 selected banks) have achieved an average positive change in pure efficiency during the study period. Only 7 banks (out of the 44 selected banks) have moderately progressed in efficiency change. In terms of scale efficiency, domestic banks have scored higher positive growth compared to that of foreign banks. The study reveals only 10 banks are scale efficient among them 9 are domestic banks. That clearly indicates that domestic banks have been successful in catching up with the economies of scale in their operations compared to that of foreign banks. During the study period, most of (8 out of 9) the foreign banks have progressed based on technical efficiency. It is notable that no domestic bank has progressed in HRM quality index. Whereas, only 2 foreign banks have progressed, namely, Standard Chartered bank and City bank NA.

Last but not least, in Table 7, the parametric t-test results disclose that foreign banks shows higher efficiency than that of domestic banks (0.946>0.833). The results also suggest that domestic banks scored as being less efficient in terms of the total efficiency, while foreign banks scored as being most efficient (0.732<0.891). Similarly, the Mann-Whitney test and Kruskall-Wallis test (non-parametric tests) also confirm the same results. Accordingly, it is of considerable importance that over the last 6 financial years, the banking sector in Bangladesh did not achieve positive growth in efficiency in any of its attributes except technical efficiency of foreign banks. Behind such operational failure in Bangladesh, the researchers identified a number of reasons: i) slow economic indicator [67], ii) high interest rate, iii) failure to achieve the targeted GDP [67], iv) capital market crisis in 2010, and v) political instability [68].

## Limitations and Future Research

The detail records of HRM practices and related investment are remain publicly unavailable to the external audiences’ use [69]. Likewise, Bangladeshi banks have limited data on HRM investment and practices. The future work in similar context should take additional variables for an in-depth analysis. In terms of pure efficiency, further study is needed to explain how pure efficiency in such a context may improve without hampering other efficiency indices? Future research can also include e-KAM model developed by Khezrimotlagh, Salleh, Mohsenpour [70] for additional discrimination in efficiency scores. Moreover, the use of panel data (e.g., generalized method of moment) can help to identify the influence of contextual variables (e.g., ownership, country governance indicator, national policy, etc.) in HRM practices and bank efficiency as developed by Da Silva e Souza and Gomes [71].

## Conclusions

This paper examines the use of traditional DEA, applying a quality index of HRM within the traditional Malmquist index model to a dataset of 44 banks of Bangladesh, from 2008–2013. We modify the conventional DEA model combining HRM as the quality index to measure bank efficiency. The superiority of this model is that it allows the calculated frontier to detect the best practice banks examining the direct impact of HRM practices.

This paper explores the importance of HRM practices in bank efficiency. In terms of pure efficiency, 8 out of 9 foreign banks in Bangladesh have found averagely progressed over the years. In total, foreign banks scored higher progress in scale efficiency compared to that of domestic banks. In view of the HRM quality index, only two foreign banks scored a positive change and not a domestic bank has found progressed over the study period. Comparing the domestic banks’ efficiency, domestic banks of Bangladesh have found progressed in terms of technical change. Most significantly, not a domestic bank has found progressed in HRM practices. The above results have also confirmed by the test of Mann-Whitney test for ensuring its robustness. The test indicates that the domestic banks are less efficient than the foreign banks in term of quality indicator of HRM practices.

This paper contributes in 4 key areas of existing literature. Firstly, it fills the literature gap by inspecting the direct effect of HRM practices by the selected 44 banks in their respective efficiencies. Secondly, the scare literature on HRM and bank efficiency especially in Bangladesh context despite its enormous importance in the international trade and business [5]. Thirdly, a comparison between the foreign banks and the domestic banks in Bangladesh demonstrates that even though the foreign banks have improved in HRM practices, they scored relatively poor efficiency scores in bank performance. Our research has driven two important implications here. First, foreign banks did not secure technical efficiency because of the existing government intervention and restricted policy [9]. Second, even if the existing HRM practices have contribution in foreign banks’ performance, foreign banks failed to secure total efficiency progress due to fail in economies of scale. The fourth contribution of this paper is model related. This paper modified the available Malmquist total factor productivity (TFP) by adding HRM as a quality component. Thus the causal relationship can be obtained through this model. The results and the findings of this paper will work as the initial off-site transmission tool for the regulators, bank managers and institutional interest groups of Bangladesh. The findings are of equal importance for both the foreign and the domestic banks in Bangladesh. It is emphasized in the discussion part that the banks within a peer group must progress in HRM practice to enhance their overall performance in terms of long-term policy implications. In addition, government of Bangladesh may consider a continuation of economic growth to allow banks to achieve scale and technical efficiency.
